# Pattern of management of urologic cancer in Saudi Arabia

**DOI:** 10.4103/0974-7796.62921

**Published:** 2010

**Authors:** Khalid Al-Othman, Naif Al-Hathal

**Affiliations:** Department of Urology, King Faisal Specialist Hospital and Research Center, Riyadh, Saudi Arabia

**Keywords:** Bladder cancer, practice pattern, renal cancer, urologists in Saudi Arabia

## Abstract

**Background:**

To compare the current uro-oncologic practice pattern in Saudi Arabia with the standard of care practice and to identify obstacles in our health care system that prevent offering such a treatment.

**Materials and Methods:**

We surveyed 247 practicing urologists in Saudi Arabia using a designed questionnaire. This questionnaire contains 19 questions focusing on management of bladder and renal cancers.

**Results:**

Of the 247 contacted urologists, 86 completed the questionnaire. Seventy six percent see more than 10 bladder cancer cases/year and 83% used rigid cystoscope for diagnosis under general anesthesia. Eighty two percent perform over 10 bladder tumor resections/year; however, 90% of them perform less than five cystectomies/year, if any. Seventy nine percent had intravesical therapy available at their hospitals and majority of them use it after resection in selected patients. Fifty percent preferred re-resection within 2–4 weeks for T1 and/or G3 tumors and majority of them (86%) perform cystectomy for muscle invasive disease and ninety six percent perform ileal conduit. Thirty four percent see over 10 renal cancers/year. Forty nine percent perform radical nephrectomy for less than 4 cm renal masses and for more than 4 cm, only 9% do laparoscopic nephrectomy while the majority preferred open technique although 77% of the hospitals participated in this survey have a urologist capable of doing laparoscopy.

**Conclusion:**

A significant number of urologists in Saudi Arabia do not apply some of the well-accepted standard practices in urologic cancer. To improve this, we need to work on our referral system and establish education and training programs to make the urologist familiar with the new modalities of treatment.

## INTRODUCTION

Urological cancer cases represent a significant and challenging part in the daily practice for the majority of urologists practicing in Saudi Arabia. So, knowing the current practice of management of urologic cancer cases inside the Kingdom may help us in different ways to improve. Bladder and renal cancers are the most common urologic cancers in Saudi Arabia. The incidence of bladder cancer in Saudi Arabia is estimated to be 201 cases/year accounting for 2.9% of all newly diagnosed cancer cases in Saudi Arabia.[1] It ranks eighth among the male population (incidence 160 cases/year) and 20th among the female population (incidence 41 cases/year). The most prevalent five regions in descending order are Tabuk 5.5/100,000, Northern region 4.5/100,000, Jouf region 3/100,000, Makkah 2.8/100,000 and the Eastern province 2.6/100,000.[1] On the other hand, renal cancer has an incidence of 198 cases/year accounting for 3.5 and 2.2% of all cancer cases/year in male and female populations in Saudi Arabia, respectively.[1]

Several reports have shown a wide variation in the practice patterns of management when compared to the guidelines with regard to diagnosis, treatment and follow-up of urologic cancers in different regions of the same country[2] or between two neighbor countries like Canada and United states.[3] The reason for conducting such a study is to compare our current practice with the standard of care practice in the developed countries which may help us to improve in certain areas and identify obstacles in our health care system that delay or even prevent offering the standard of care treatment to urologic cancer patients.

## MATERIALS AND METHODS

A survey on bladder and renal cancers management was conducted by distributing a questionnaire to 247 urologists practicing in Saudi Arabia.

The questionnaire was distributed either:

1. directly during several educational activities, for example, urology club meeting, workshops and the annual Saudi Urological Association meeting.
2. electronically through an email which has a simple online questionnaire that can be found on http://appforms1.kfshrc.edu.sa/Forms/bladder.nsf/Bladder?OpenForm

The questionnaire was sent to urologists working in different health sectors in Saudi Arabia like university, military, National Guard, security force, Ministry of health, private and specialist hospitals. It contains 13 questions on bladder cancer and 6 on renal cancer.

## RESULTS

Out of 247 urologists, only 86 responded, with a response rate of 35%. Four responses were excluded because of multiple answers; therefore, only 81 urologists were included in this analysis. Each question and its response are shown below.

**Question 1: How many bladder cancer cases you see per year?**

Seventy six percent see more than 10 cases per year, seventeen percent see 5–10 cases and the rest see less than 5 cases per year.

**Question 2: What method of endoscopic examination you use to diagnose bladder cancer?**

Eighty three percent use rigid cystoscope and the remaining 17% use flexible cystoscope.

**Question 3: What type of anesthesia you use during cystoscopic examination?**

Majority of them use general anesthesia (88%), whereas (12%) use local anesthesia.

**Question 4: How many bladder tumor resections you perform each year?**

Eighty two percent perform more than 10 procedures per year, 11% perform 5–10 cases and 7% perform less than 5 cases per year.

**Question 5: How many radical cystectomy cases you perform each year?**

Ninety percent of them perform less than five procedures, if any, per year and 10% perform 5–10 cases.

**Question 6: How many radical cystectomy cases are performed in your hospital each year?**

Seventy six percent of the hospitals perform less than 5 cases per year whereas 20% perform 5–10 cases and the remaining 4% perform more than 10 cases per year.

**Question 7: Is intravesical BCG and/or mitomycin available at you hospital?**

Seventy nine percent have it available at their hospital, whereas 21% do not have it.

**Question 8: Is intravesical therapy prescribed by urologists?**

Ninety one percent answered yes and 9% responded no.

**Question 9: After bladder tumor resection, do you give or refer patient for intravesical therapy in certain conditions only?**

Eighty eight percent give only in certain conditions, whereas the other 12% give to all.

**Question 10: After bladder tumor resection, do you have a standard protocol for follow up?**

Ninety six percent have, whereas 4% do not have.

**Question 11: After bladder tumor resection, if the tumors if T1 and/or G3, do you consider re-resection within 2 to 6 weeks?**

Forty seven percent only do, whereas 53% do not.

**Question 12: After bladder tumor resection, if the tumor is muscle invasive your next step is?**

Radical cystectomy in 86%, 11% refer the patient and the remaining 3% refer to radiation oncologist.

**Question 13: After radical cystectomy, the most common method you perform for urinary diversion is?**

Ninety six percent perform ileal conduit, 2% orthotopic bladder and 2% continent urinary diversion.

**Question 14: How many renal cancer cases you manage per year?**

Forty four percent see less than 5 cases per year, 40% see 5–10 cases and the remaining 16% see more than 10 cases per year.

**Question 15: How many renal cancer cases you see in your hospital per year?**

Twenty six percent of the hospitals manage less than 5 cases, 40% manage 5–10 cases and 34% manage more than 10 cases per year.

**Question 16: What will you do for renal masses equal or less than 4 cm?**

Forty nine percent perform open radical nephrectomy, 7% perform laparoscopic radical nephrectomy and 44% perform open partial nephrectomy, whereas none do laparoscopic partial nephrectomy.

**Question 17: What will you do for renal masses more than 4 cm?**

Ninety one percent do open radical nephrectomy, whereas the other 9% do laparoscopic radical nephrectomy.

**Question 18: Which approach you think is better for renal masses equal to or more than 4 cm?**

Sixty nine percent think open approach is better and only 31% prefer laparoscopic approach.

**Question 19: At your hospital, do you have an urologist perform laparoscopic nephrectomy procedure?**

Seventy seven percent answered yes, whereas 23% lack this.

## DISCUSSION

Comparing the current practice for the treatment of urologic cancer cases in Saudi Arabia, as reflected in this questionnaire, with the standard practice applied in centers of excellence and/or recommended in the official guidelines like the American Urological Association (AUA), European Association of Urology (EAU), American Society of Clinical Oncology (ASCO) and National Comprehensive Cancer Network guidelines, we can observe that the following procedures are not applied as commonly as expected: laparoscopic nephrectomy (9%), nephron sparing surgery for small renal masses (44%), a re-resection for bladder tumors T1 and/or G3 (47%), diagnostic flexible cystoscopy (17%) and orthotopic new bladder (2%).

Laparoscopic radical nephrectomy may now be considered a standard of care for patients with T1–T2 renal cancer because intermediate data indicate equivalent cancer-free survival rates and a lower morbidity when compared with the open approach;[4‐6] despite this fact, only 9% of our questionnaire responders have preferred to perform this procedure for renal masses equal to or larger than 4 cm, whereas the majority still prefer open radical nephrectomy. Kaynan *et al*. have shown that very few urologists use laparoscopy, in a survey of urological laparoscopic practices in the state of California, a finding that is consistent with data from the AUA directory survey and they relate this to the fact that indications are largely a function of individual technical ability. Urologists being not adequately trained during residency is a major limitation of current laparoscopic practice.[7] Duchene *et al*. have surveyed 1056 urology residents and concluded that residents are participating in most cases of urologic laparoscopic surgery but only 38% consider their laparoscopic experience to be satisfactory. A need still exists for increased laparoscopic training for residents, which can be accomplished by expanding the training facilities and increasing the number of faculty members performing laparoscopic procedures.[8] We think that besides the deficiency of laparoscopic training courses in Saudi Arabia, we have a low level of awareness of the value of this minimally invasive modality.

Less than half of the urologists perform partial nephrectomy for small renal masses although nephron sparing surgery, when performed in patients with a solitary tumor less than 4 cm, provides recurrence-free and long-term survival rates similar to those observed after radical surgical procedures.[9‐11] Nephron sparing surgery is an established curative approach for the treatment of patients with renal cell carcinoma.[10,12] Van Poppel showed in an EORTC intergroup phase 3 trial that radical nephrectomy patients had a median follow-up serum creatinine of 1.5 mg and a mean of 1.6 mg (minimum 0.89; maximum 4.74), whereas nephron sparing surgery patients had a median follow-up serum creatinine of 1.29 mg and a mean of 1.34 mg (minimum 0.78; maximum 4.55). The values are significantly lower on nephron sparing surgery (*P* < 0.0001).[13] Diabetes is an endemic disease in Saudi Arabia with an increasing prevalence from 17 to 25% over 10 years.[14] In a country where diabetic nephropathy is so frequent, the preservation of nephron mass advances from a desirable state more to a necessity. The 5-year survival rate for patients who had diabetes and started renal replacement therapy in 1955 was 33.6%.[15] From this viewpoint, the survival of this group is worse than the survival rate in many malignancies and if radical nephrectomy is done and the other kidney fails because of preexisting renal condition, the resulting end-stage renal disease could have more devastating outcome on the patient's medium and long-term survival than the rarely observed occurrence of metastasis following nephron sparing surgery.[16]

As a followup for this study, when we did an analysis with the urologists not performing partial nephrectomy for renal masses less that 4 cm, for the possible reasons behind this, majority claimed that fear of complications, lack of proper medical and surgical backup, lack of experience and training to perform this kind of operations, cancer control issues and technical complexity are the main reasons.

According to the European association of urology guidelines, a re-resection of bladder tumor after 2–6 weeks should be performed if incomplete resection was done, lack of muscle tissue in the specimen or detection of T1 and/or high grade tumor in the initial resection because it has been demonstrated that second re-resection leads to reduced recurrences and improved prognosis.[12] Despite this, still a majority of the responses declined this.

Konety *et al*. have observed that hospitals with an average volume of greater than 7.5 cystectomies per year had lower mortality;[17] in the current study about 90% of the urologists perform less than five radical cystectomy cases, if any, per year which we think is too little a number to master such a major procedure. Harry Herr stated that *"experienced urologic oncologist operating in high volume centers tend to achieve better survival results than urologic surgeons who perform few cases in low volume institution."*[18] Eighty eight percent of urologists in Saudi Arabia prefer to give intravesical chemotherapy only in certain conditions, for example, with multiple recurrences, although several studies have shown a significant recurrence-free survival when given after each resection of bladder tumor.[12,19] Despite the wide availability of flexible cystoscope for surveillance of bladder tumor under local anesthesia, 83% of urologists in the kingdom use rigid cystoscope mostly with general anesthesia in 88% of instances. Wright *et al*. reported a comparable prevalence that 80% of instances a rigid cystoscope was used and concluded that the cost of the flexible cystoscope over the rigid cystoscope was the main reason.[20]

These variations in the current urologic practice in Saudi Arabia are possibly attributed to certain factors which delay or prevent applying these procedures in the management of urologic cancer in Saudi Arabia.

One of these factors is lack of awareness of the value of these modalities, for example, partial vs. radical nephrectomy. Another one is tendency of urologists to perform surgical operations that they are familiar with and could be done safely at their hospitals, for example, ileal conduit vs. orthotopic new bladder. Also, another factor is a lack of educational and training programs which help urologist to be familiar with these procedures, for example, laparoscopic vs. open nephrectomy. There is another issue which can give additive effect indirectly like the administrative difficulties in the referring system from primary to secondary or secondary to tertiary health care facilities, In addition, the urologist himself may be reluctant to refer such cases and thus it has been shown by Joudi *et al*. in the United States that up to 81% of urologists were reluctant to recommend cystectomies because intravesical therapy in community practice conforms with the generally accepted indications for high-grade and T1 disease; however, the presence of carcinoma *in situ* (CIS) with high-grade T1 disease seems to drive preferences toward radical treatment instead of intravesical immunotherapy.[21]

## CONCLUSION

This study showed that significant number of urologists in Saudi Arabia do not apply some of the well-accepted surgical procedures in the management of urologic cancer. Improvement of referral system between our health care facilities may give cancer patient a better chance to receive the optimum treatment. Establishing and increasing educational and training activities can make the urologists more familiar with the new modalities of treatment and encourage them to apply these modalities in their practice particularly those which are standard of care.
